# Homocysteine, Cobalamin and Folate Status and their Relations to Neurocognitive and Psychological Markers in Elderly in Northeasten of Iran

**Published:** 2013-06

**Authors:** Lida Manavifar, Habibollah Nemati Karimooy, jamshid Jamali, Morteza Talebi doluee, Abbas Shirdel, Amireh Nejat shokohi, Mahdie Fatemi nayyeri

**Affiliations:** 1 Paramedical School, Mashhad University of Medical Sciences, Mashhad, Iran; 2 Department of Neurology and Physiology, School of Medicine, Mashhad University of Medical Sciences, Mashhad, Iran; 3 Department of Biostatistics, School of Medicine, Shiraz University of Medical Sciences, Shiraz, Iran; 4 Department of Internal Medicine, School of Medicine, Mashhad University of Medical Sciences, Mashhad, Iran; 5 Assistant Professor, Dept. of Emergency Medicine, School of Medicine, Mashhad University of Medical Sciences, Mashhad, Iran; 6 Graduated of Birjand University of Medical Sciences, Mashhad, Iran

**Keywords:** Cobalamin, Folate, Homocystein, Psychological, Neurocognitive, The elderly

## Abstract

***Objective(s):*** Incidence of neurocognitive and psychological disorders may be related to serum homocystein (Hcy), cobalamin (vitamin B_12_) and folate levels in old people. The aim of this study was to assess the relation between Hcy, cobalamin, folate and neurocognitive and/or psychological disorders in the elderly.

***Materials and Methods:*** In this cross-sectional study, 280 subjects with ≥ 65 years old ,were evaluated. The subjects were selected from 12 regions of Mashhad, Iran, over March to October 2009. After blood sampling, data were collected by questionnaire, face to face interview and performing neurocognitive and psychological tests. The sera of 250 persons were analyzed for cobalamin and folate by RIA method. Amongst the aforementioned samples, 78 cases with cobalamin <300 pg/ml and folate <6.5 ng/ml were analyzed for Hcy by ELISA method.

***Results:*** Amongst the people, 126 (45%) were male and 154 (55%) were female. The prevalence of hyperhomocysteinemia (HHcy) was 59.5% and 37.1% in male and female respectively (*P* -value =0.049). Hcy inversely correlated to cobalamin (r=-0.282, *P*=0.014) and to folate (r=-0.203, *P*=0.014). Hcy, cobalamin and folate correlations to neurocognitive and psychological impairments were not statically significant.

***Conclusion:*** Hyper Hcy or low cobalamin and folate in the elderly, are prevalent but their relationships with neurocognitive and psychological impairments is controversial. If these relationships had been confirmed, performing a single serum Hcy or cobalamin test would have been enough enough to diagnose and prevent neurocognitive impairments and inversely, neurocognitive-psychological sign and symptoms could have meant probable tissue vitamin deficiencies. However methods of assessing neurocognitive and psychological markers with validity and reliability of clinical and laboratory tests for finding aforementioned relationships should be revised.

## Introduction

Annually many old people refer to physicians or clinics with mild and vague symptoms such as anemia and neurocognitive and psychological impairments that may be due to cobalamin and folate deficiency followed by gastric atrophy and malnutrition.

Two enzymatic reactions dependent on cobalamin (Cbl), has been identified of which one converts methylmalonyl–coenzyme A to succinyl-CoA using adenosyl-cobalamin as a cofactor. So Cbl deficiency will increase methylmalonyl-CoA as methylmalonic acid (MMA). The other enzyme reaction has role in the synthesis of methionine from homocysteine (Hcy) using methyl-cobalamin as a cofactor whicht is important for definition the neurocognitive and/or psychological aspects of cobalamin and folate deficiency. Both Deficiencies involve in production of tetra Hydrofolaet and cause megaloblastic changes in red blood cells (1).

The common symptoms of cobalamin and folate deficiency are anemia, macrocytosis and neurologic symptoms such as parenthesis, ataxia, dementia, depression and psychosis (2). Frequently neurologic progression occurs before hematologic symptoms( 3 ).

Hyper homocysteinemia is caused by various conditions including cobalamin and folate deficiency, renal failure, genetic defects, decreased blood volume and hyperthyroidism. These are independent risk factors for nervous degeneration and cardiovascular and cerebrovascular disorders in old people and thus require special medical management (4 -6 ). Patients with vascular disease have higher plasma levels of homocysteine than patients without vascular disease which indicates vascular risk factors may play a role in the development of cognitive impairments ( 7 ).

Cobalamin and folate deficiencies are involved in pathogenesis of cognitive status in old people through hyper homocysteinemia. High levels of homocysteine due to vitamin B deficiency (folate, vitamin B_12_ and B_6_ whicht are required in methylation of homogystein to methionin) correlate with decline cognitive performance ( 8 ). However vitamin B supplementation influences are controversial and only a few cognitive tests have shown statistically significant improvements ( 9 ).

Up to 15% of old population in the United States and Europe have elevated methylmalonic acid that is associated with low or borderline levels of serum cobalamin( 10 ). Folate deficiency in Sweden was reported up to 20% ( 11 ). In a survey in low income Population in South West region of Tehran, Iran, age-adjusted incidence of low serum cobalamin was 27.2% in female and 26.32% in male. Moreover, low serum folate level was 97.92% in female and 98.67% in male ( 12 ). A study in United Kingdom showed increasing deficiency from about 1 in 20 among people aged 65-74 years to 1 in 10 among people aged 75 years or older ( 5 ). 

In the past 10 years, studies have shown high incidence of neurocognitive and/or psychological disorders in Iran ( 13 ). In a psychiatric interview on a sample of Iranian population at the age 18 and older, the prevalence of psychiatric disorders was 10.81%. The prevalence of anxiety and mood disorders were 8.35% and 4.29%, respectively. Prevalence of psychotic disorders was 0.89%, neurocognitive disorders 2.78% and dissociative disorders 0.77 % ( 14 ). According to geriatric depression scale (GDS), 23.5% of old people (60-98 years old) living in Razavi Khorasan province in Iran, are at risk of depression ( 15 ).

Regard to the prevalence of neurocognitive and psychological impairments, hyperhomocysteinemia, cobalamin and folate deficiency in the elderly, assessments for finding relationships between mentioned deficiencies and impairments have been performed, which showed controversial results. By confirming aforementioned relationships, diagnostic and preventative operations might be suggested for the improvement of neurocognitive-psychological impairments in old peoples. Well maintenance of cognitive ability in the elderly is vital not only to provide suitable health status but also to retard the onset of dementia, prevent its progression, increase productivity and decline societal costs for taking care of these people ( 8 ).

## Material and Methods

In this cross-sectional study with cooperation of statistic unit of Khorasan province health centre, 280 male and female, over 65 years old, were selected amongst twelve regions of Mashhad. Seven statistic questioner students selected to call and obtain written informed consent to participants and fill a demographic questionnaire. 250 cases were referred to central laboratory in Emam Reza hospital on March to October 2009. Medical history, medications, smoking habits, vitamin supplement uptake and lifestyle habits were taken from all participants in a face-to-face interview by a practitioner. Also physical and clinical sign and symptoms and results of neurocognitive and psychological tests Including mini-mental state exam (MMSE), deep tendon reflex (DTR), Romberg test, geriatric depression scale (GDS), insomnia, motivation, easily crying, pain perception and also persons documents for dementia were recorded in a special form.

Exclusion criteria in participant were creatinine>1.5 mg/dl, B vitamin Supplementation (vitamin B_12_, B6 and folic acid) in recent 3 months, smoking, medication with metformin ,trimetoprim and omeprazole. Elderly with more than 65 years and completed data in questionnaires and forms were included in our study. After blood sampling, sera were collected and kept in -20 ˚C until measuring. Serum cobalamin and folate analyzed by RIA method (DRG kits Cat#, RIA-1990, Germany) Serum methylmalonic acid (MMA) often increases with cobalamin deficiency while homocysteine increases with both folate and cobalamin deficiency, conclusively assaying of serum homocysteine was preferred (16-17). 78 cases, who had cobalamin <300 pg/ml and folate< 6.5ng/ml and also no confounders were analyzed for homocystein by Elisa method (DRG kit Cat#: EIA-2925, Germany).

According to a study on the same population by authors, a cut off point <330 pg/ml and <6.5 ng/ml was selected as cobalamin and folate deficiency respectively. In the same study, hyperhomocysteinemia defined as >15 µmol/l.

***Statistical analysis***

Statistical analysis was conducted using the SPSS version 11.5. Normally distributed quantitative variables were demonstrated as mean ± standard deviation. The normality condition of the quantitative variables was investigated by using the Shapiro-Wilks test. Mann-Whitney and Kruskal-Wallis Tests were used to compare the means of serum cobalamin, folate and homocystein in 

**Table1:** Average distribution of serum cobalamin, folate and homocystein by sex

		N (% )	Mean (SD)	*P*-value
Cobalamin(pg/ml)	Total	244	299.92 (374.19)	0.001
	Men	107 (43.85%)	244.74 (232.28)	
	women	137 (56.15%)	343.020 (451.51)	
Folate( ng/ml)	Total	241	5.31 (3.27)	0.012
	men	107 (44.40%)	4.96 (3.47)	
	women	134 (55.60%)	5.60 (3.08)	
Homocystein (µmol/L)	Total	77	16.40 (8.06)	0.015
	men	42 (54.55%)	18.52 (8.81)	
	women	35 (45.45%)	13.86 (6.27)	

independent variables (sex, MMSE, DTR tests, Romberg, geriatric depression scale, vibration, pain perception, insomnia, motivation and easily crying, and dementia). Pearson coefficient of correlation was used to assess relation between serum cobalamin, folate and homocystein. P-value of less than 0.05 was considered as significant.

## Discussion

The aim of this study, carried out prospectively in an urban elderly population in North East of Iran, was to discovery relationships between neurocognitive psychological impairments and three components of serum: cobalamin (vitamin B_12_), folate and homocystein. Previous studies had shown high prevalence of hyperhomocysteinemia (HHcy), cobalamin and/or folate deficienciy and cognitive impairments in the elderly (4,10-11, 18-19). In Eastern Indians and in Iran HHcy has high prevalence, (44.6% and 41.07%) while 17% and 9.5% of population in California and Finland, suffer from this condition respectively (12, 20). Carmel *et al* reported its prevalence 26.1% for people without renal disorders while half of them had been identified with low cobalamin or folate levels (29 ).

A study on old population in North East of Iran, with respect to cut off points, showed that the prevalence of cobalamin deficiency with low levels (<122 pg/ml) was 22.7% and with borderline levels (122-330 pg/ml) was 51.8% and the prevalence of folate deficiency with low levels (<3 ng/ml) was 16.7% and with borderline levels was (<6.5 ng/ml) 64.2 % (21).

Previous studies have shown correlation between low level of folate and cognitive impairments. The therapeutic response of Alzheimer's disease to cholinesterase inhibitors is improved by folic acid supplementation (8, 22). Patients with Fluoxetine-resistant major depression disease (MDD) were found to have low serum level of folate (23). Foltein`s minimental test has shown 45% of the elderly with low cobalamin levels have mental disorders (24). In a study by Shahar *et al, 640 *patients were studied. 50% of old people present in the population had low or borderline levels of serum cobalamin (120-150 pmol/L) which correlates to both cerebrovascular diseases and cognitive disorders (*P*=0.046) (19). Incidence of vitamin B_12 _and folic acid deficiency in Venezuelan old people was 26.4% and 43.4% respectively. Also with Foltein`s minimental test, 49% of the elderly had mental disorders and vitamin B_12_ levels were significantly lower in this group (4). A study has reported that 28% of patients with dementia have high level of serum homocysteine while the level of serum methylmalonic acid (which increases as a result of cobalamin deficiency) was normal (25).

Normal serum homocystein has been defined as<14 µmol/l (26), or <15 µmol/l (15, 27). Serum methylmalonic acid (MMA) is often increased with cobalamin deficiency while homocysteine increases with both folate and cobalamin deficiency, so assaying of serum homocysteine was preferred (16-17). In our study, prevalence of HHcy was 49.4% (59.5% of men and 37.1% of women) which was in agreement with previous studies in different populations (28). Like other studies (21, 29) serum homocysteine correlated inversely with serum cobalamin and folate (*P* = 0.001 for both).

***Cut off points for cobalamin and folate deficiency***

Many researchers has determined cut off points for cobalamin and folate deficiency. Figlin et al defined cobalamin deficiency as <220 pg/ml with MMA >0.24 µ mol/l, and folic acid deficiency as <4.9 ng/ml with homocysteine >15 µ mol/l (30) and Rajan et al defined cobalamin deficiency equal to 299 pg/ml (31). Walters et al reported elevated MMA and homocysteine associated with cobalamin <350 pg/ml and folate level < 3 ng/ml as cut off point in the elderly women (16). On the basis of a previous study on the participant of this study, we select cut off point <330 pg/ml and <6.5ng/ml for cobalamin and folate deficiency respectively (21). 

***Correlation between cobalamin and folate deficiency and HHcy***

Our study showed significant association between serum cobalamin and folate (r=559, *P*≤0.001) and a negative association between HHcy and cobalamin (r=-0.282, *P*=0.014) and folate (r=-0.203, *P*=0.014).

***Correlation between HHcy, cobalamin and folate deficiency with neurocognitive impairments***

HHcy and Cobalamin and folate deficiency are associated with cognitive impairments and dementia in the elderly. They often ingest cobalamin less than 1 µg/day(less than Recommended Dietary Allowance =2-4 µg/d). Prevalence of HHcy with this Regimen is reported(10). As cognitive impairments are often incurable, investigation of their modifiable risk factors such as HHcy, cobalamin and folate deficiency is recommended (8). 

Correlation of HHcy or cobalamin deficiency with neurocognitive dysfunctions is controversial. It is unknown whether HHcy or vitamin deficiency is responsible for neurocognitive impairments (32). 

*Lidballe et al* observations on 839 old people have shown that score of cognitive functions as measured by mini-mental state examination and low wellness have significant corralations with markers of cobalamin insufficiency such as low serum cobalamin, high methylmalonic acid, high homocysteine but their causatives are uncertain (28). The findings of *Nilsson et al* implied that elevated plasma Hcy in old patients with mental illness is mainly associated with the presence of vascular disease and is not related to any specific psychiatric diagnosis (33). *Stabler et al *found Positive relations but *Hvas et al* and *Negga et al* didn’t found any relations between serum methylmalonic acid and neurologic and gastrointestinal symptoms (25, 27, 34). Homocysteine is negatively correlated with neuropsychological scores, but there are little evidences to justify treating cognitive impairment with cobalamin or folate supplement in peoples over 60 years old (25). However one study has shown that folic acid improve therapeutic response of people with Alzheimer's disease while a few other studies have indicated no effect on cognitive functions (22). Dolatabadi *et al* showed that folic acid have therapeutic and preventive effects on cognitive impairments and improves memory performance and learning in Alzheimer's disease (38).

***Correlation between Serum HHcy, cobalamin and folate deficiency and dementia***

In our study, 20.8% (15 persons) of the elderly with cobalamin <300 pg/ml and folate <6.5 ng/ml and HHcy had dementia. In these patients correlation between dementia and cobalamin, folate deficiency and HHcy was statically insignificant, but elderly with dementia had higher homocystein serum levels (17.91±8.18 µmol/l) than elderly without dementia. 

***Correlation between Serum Hcy, cobalamin , folate and*** ***MMSE test***

 MMSE (Mini mental state examination) is positive in mental disorders. Normal score is >20. In our study serum Hcy, cobalamin and folate levels in two groups of positive and negative MMSE test patients had no significant differences, but the elderly with positive MMSE test had higher Hcy level than negative group. This was not in contrast with the findings of Ellison* et al *(35). Kado* et al* in 74-79 years old Population (n=880) in 10 European Country found low but significant relation between MMSE score and cobalamin and also Homocysteine (32). 

***Correlation between Serum Hcy, cobalamin, folate and*** ***geriatric depression scale*** (***GDS)***

Our results showed that serum Hcy, cobalamin and folate average in the 2 groups with positive (score>5) and negative (score≤5) geriatric depression scale test have not significant differences. The same insignificance was observed with Motivation, Insomnia and easily crying.

***Correlation between Serum Hcy, cobalamin, folate and*** ***deep tendon reflex*** ***(DTR), Romberg test***

We investigate relations between Hcy ,cobalamin, folate, and Neurocognitive Markers by deep tendon reflex test and Romberg test. In two tests, relations were insignificant but the elderly with positive and high scores in DTR test had higher homocystein (18.41 µmol/L) than negative. The same insignificant differences were observed with Vibration and Pain perception.

Consistent to results in some studies, we showed that there are not significant relations between neurocognitive-psycholigical impairments and hyperhomocysteinemia or cobalamin and folate deficienciy in the elderly.

Correlations between laboratory and clinical markers for these disorders in various studies are heterogenous and it is reasonable to assume that there are multi factors resulting in these variations including: different etiologies, stage of the disease, degree of manifestation of symptoms, sampling representative the entire population, diversity of tests and measurement techniques. Normal values for serum cobalamin and its metabolites is not established and various criteria, tests, and cutoff points have been used to define cobalamin deficiency (36). Also minimum value that disturbs nervous system functions is not yet recognized (20). Some studies indicate that vitamin B supplementation normalizes plasma *Hcy* and *MMA* (methyl malonic acid) levels without any improvement on cognitive performance and they only delay progression of the disease. This may be due to an insufficient dose and duration of supplements, irreversible or vitamin-independent neurocognitive impairments (8, 18, 37, 38).

In this study we carefully collected participant data by biochemical tests and questionnaires and this was probably more accurate than neurocognitive and psychological examinations. It seems these clinical or cognitive tests are not adequate or sensitive enough to detect mild dysfunctions in these disorders.

 These problems are consistent to Bjorkegren *et-al* explaining that since the Nerocognitive disorders are the first symptoms of cobalamin and folate deficiency, their diagnosis need much qualified instruments and their investigations require enough subjects and appropriate composition of population and correct sampling (11). Christopher *et al * refer to the sensitivity and specificity of metabolite measurements for milder deficiency status that are uncertain (8).

## Conclusion

Due to prevalence of neurocognitive and psychological impairments, hyperhomocysteinemia ,cobalamin and folate deficiency in the elderly, finding crucial relationships between mentioned impairments and deficiencies, which seem to be controversial, is essential. By confirming aforementioned relationship, diagnostic and preventative operations could be initiated to improve neurocognitive- psychological impairments for desired peoples. So primarily, methods for assessment of neurocognitive and psychological status and validity and reliability of clinical and laboratory tests should be revised and secondly, longitudinal studies and clinical trials should be performed in old population to determine whether lowering of homocysctein or increasing cobalamin and folate levels will improve neurocognitive psychological impairments or not.
